# Sex-dependent effects of short- and long-term social isolation on behavior and medial prefrontal DAT in rats

**DOI:** 10.1038/s41598-026-48488-1

**Published:** 2026-04-27

**Authors:** Negin Marghoob, Batool Ghorbani Yekta

**Affiliations:** 1 Department of Physiology, Faculty of medical science, Islamic Azad University, Tehran, TeMS.C Iran; 2 Cognitive and Neuroscience Research Center (CNRC), TeMS.C, Islamic Azad University, Tehran, Iran; 3 Applied Biotechnology Research Center, TeMS.C, Islamic Azad University, Tehran, Iran; 4 Herbal Pharmacology Research Center, TeMS.C. Islamic Azad University, Tehran, Iran; 5 Tehran Medical Branch, Department of Physiology, Faculty of Medical Science, Islamic Azad University, TeMS.C, Tehran, Iran

**Keywords:** Social isolation, Sex difference, Locomotor activity, OCD-like behavior, Anxiety-like behavior, Dopamine transporter (DAT), Neuroscience, Psychology, Psychology

## Abstract

Dopamine transporter (DAT) via affecting dopaminergic signaling may modulate behavioral alterations in various stressful conditions. The present research aimed to assess the potential deleterious effects of social isolation on behavioral functions and DAT levels in the medial prefrontal cortex of both sexes of rats. Social isolation was done in two protocols: 25 days (PND45-70) and 50 days (PND20-70). Open field test, hot plate, marble burying test, forced swim test, and western blotting were used. The results showed locomotion, grooming, and climbing were increased in both protocols of social isolation in both sexes. Anxiety-like behavior in the open field test was increased in both protocols of social isolation in males, while in females, only short-term social isolation showed this effect and long-term social isolation decreased it. Sniffing was decreased in both protocols of social isolation in males, while in females, only long-term social isolation increased it. Rearing was decreased in both protocols of social isolation in males, while in females, only long-term social isolation decreased it. Pain threshold was decreased in both protocols of social isolation in both sexes. OCD-like behavior was increased in both protocols of social isolation in both sexes. Immobility was only decreased in socially isolated female rats. Overall, the findings suggest that social isolation is associated with sex- and protocol-dependent behavioral alterations accompanied by changes in DAT protein levels in the medial prefrontal cortex. In sum, we showed the potential role of DAT in the deleterious effects of social isolation, with sex- and duration-dependent effects of social isolation and DAT.

**Supplementary Information:**

The online version contains supplementary material available at 10.1038/s41598-026-48488-1.

## Introduction

Social isolation is widely regarded as a serious stressor for social animals such as rats and mice. Many studies have used a social isolation model to induce behavioral dysfunctions and cognitive impairments in rodents^1–3^. Also, multiple lines of work indicate that social isolation may be considered as a model to induce schizophrenia-like model in rodents^4,5^. It has also been revealed that social isolation in rodents leads to altered locomotor activity, and manic-, depressive-, anxiety-, and OCD-like behaviors^6–9^. Social isolation dramatically affects the function of proteins involved in promoting synaptic plasticity and neurogenesis such as the brain-derived neurotrophic factor (BDNF) and cAMP response element-binding (CREB), increases neuroinflammation, and increases apoptosis^8,10–15^, underlying cognitive deficits and mood disturbances. Of note, social isolation can significantly affect dopaminergic signaling^16,17^, a mechanism that is involved in the induction of schizophrenia-like behavioral phenotypes, manic-like behavior, and hyperlocomotion^18–20^.

As we know, dopamine is a critical neurotransmitter with a wide range of important functions including the modulation of movement, motivation, pleasure, and reward^21^. Evidence has shown that the mood state is significantly affected by the level of dopamine in the synaptic cleft or the level of dopamine release^22,23^. It has been reported that deficits in dopaminergic signaling are closely related to different psychiatric conditions, such as schizophrenia and depression^24,25^. It has also been shown that changes in dopaminergic signaling in the hippocampus may be related to Huntington’s diseases^26^, Parkinson’s disease^27^, attention deficit hyperactivity disorder (ADHD)^28^, and Alzheimer’s disease^29^. As mentioned, social isolation can also affect dopaminergic signaling, leading to mood disturbances and cognitive deficits^30,31^.

The dopamine transporter (DAT), also known as SLC6A3, is a protein that reuptakes dopamine from the synaptic cleft, leading to the regulation of dopaminergic signaling^32^. DAT acts as a transporter protein that actively transfers dopamine from the synaptic cleft back into the presynaptic neuron^33^. Evidence has shown that any dysregulation in the function of DAT is associated with several psychiatric conditions, including Parkinson’s disease, schizophrenia, depression, and addiction^32^. Reports indicate that chronic unpredictable mild stress-induced depression in rats is associated with increased DAT levels in the prefrontal cortex^34^. It has been declared that stimulation of dopaminergic signaling via administration of chronic pramipexole in a rat model of Parkinson’s disease is involved in its’ antidepressant effect, with no effect on DAT^35^. Further, a related study has found that alcohol consumption increases DAT sites in different brain regions in Wistar-Kyoto (WKY) rat (a rat model of depression)^36^. On the other hand, Prior work shows that DAT knock-out rats show increased locomotor activity and decreased sociability, the symptoms like those seen in schizophrenia^37^. Importantly, social isolation has been shown to increase DAT and the susceptibility to psychostimulants in female rats^38^. Previous study has concluded that elevated DAT may be a suggested mechanism by which social isolation leads to enhanced stimulant potencies and reinforcing properties^39^. Social isolation also leads to lasting increases in anxiety-like behaviors, dopamine releases, and DAT activity^40^. The medial prefrontal cortex plays a central role in emotional regulation, executive functions, and dopaminergic modulation, making it a critical region for examining DAT alterations following social isolation^41^. Thus, it seems that DAT function may be an important underlying mechanism for mood disturbances and cognitive deficits induced by social isolation. Social isolation may exert distinct neurobehavioral effects depending not only on its duration but also on the developmental stage at which isolation begins. Notably, the postnatal period between PND20 and PND45 represents a critical window for medial prefrontal cortex (mPFC) maturation, characterized by ongoing synaptic refinement, dopaminergic innervation, and development of emotional regulation circuits. Disruption of social input during earlier developmental phases may therefore induce long-lasting alterations in dopaminergic signaling and emotional processing, whereas isolation commencing after partial maturation of the mPFC may induce comparatively attenuated or differential outcomes. Accordingly, we hypothesized that earlier initiation of social isolation (PND20–70) would produce more pronounced and persistent alterations in dopamine transporter (DAT) expression and affective behaviors than isolation initiated during late adolescence (PND45–70), with potential sex-dependent vulnerability. This design allows differentiation between the effects of isolation duration and the timing of exposure during neurodevelopment^42^.

The behavioral test battery was selected to assess complementary functional domains that are known to be modulated by dopaminergic signaling, including locomotor activity, anxiety-like behavior, compulsivity-related behavior, stress-coping strategies, and nociceptive processing. This multidimensional approach allowed an integrated evaluation of behavioral disturbances induced by social isolation across different developmental periods and between sexes^30^. Accordingly, the present study examined the effects of two social isolation protocols initiated at different developmental stages on behavioral outcomes and DAT protein levels in the medial prefrontal cortex of male and female rats.

## Materials and methods

### Animals

Forty-two Wistar rats [21 males and 21 females, bred in Cognitive Neuroscience Lab with a 12/12 h circadian cycle and normal temperature (22 ± 1 °C)] were divided into six different experimental groups, while 7 rats (each group) were housed per cage (25*50*20 cm) with *ad libitum* access for food and water. Estrous cycle stages were not monitored, and female rats were tested under freely cycling conditions. This approach was chosen to reflect natural hormonal variability; however, it may contribute to increased variability in behavioral responses. This limitation is acknowledged and discussed^43^. Although estrous cycle stages were not synchronized, randomization of females and conducting all tests within the same circadian window minimized systematic hormonal bias. Similar sample sizes have been widely used in comparable behavioral neuroscience studies^44^. Some rats were used for short-term social isolation (25 days) at the age of PND45 and other rats were used for long-term social isolation (50 days) at the age of PND20. The onset of social isolation was selected based on the developmental trajectory of the dopaminergic system. PND20 corresponds to the post-weaning and juvenile period, during which mesocortical dopaminergic projections, synaptic pruning, and receptor organization within the medial prefrontal cortex are highly dynamic and sensitive to environmental influences. Social deprivation during this developmental window has been shown to induce persistent alterations in dopamine uptake, release dynamics, and stress responsivity. In contrast, PND45 represents late adolescence, a stage in which dopaminergic circuitry is structurally more mature but remains functionally plastic. By terminating both isolation protocols at PND70, the present study aimed to distinguish the effects of developmental timing from the duration of social isolation on behavioral outcomes and DAT protein expression^38^. The control rats were at the age of PND70. The tests for socially isolated rats were also done at the age of PND70. The tests were done during 8 a.m. to 12 p.m. The design of the experimental groups and behavioral procedures have been done in accord with National Institutes of Health Guide for the Care and Use of Laboratory Animals^45^. The study is fully reported in accordance with the ARRIVE guidelines, ensuring transparent and comprehensive reporting of animal research methodologies.

### Experimental groups

This study consisted of 6 groups (*n* = 7):

Group 1- Male control: male rats remained intact.

Group 2- Female control: female rats remained intact.

Group 3- Male short-term social isolation: male rats were socially isolated for 25 days (PND45-70).

Group 4- Female short-term social isolation: female rats were socially isolated for 25 days (PND45-70).

Group 5- Male long-term social isolation: male rats were socially isolated for 50 days (PND20-70).

Group 6- Female long-term social isolation: female rats were socially isolated for 50 days (PND20-70).

The order of the tests was as follows: open field test, hot plate, marble burying test, forced swim test, and western blotting to evaluate dopamine transporter (DAT) protein levels in the medial prefrontal cortex. There was an interval of 60 min between open field test and hot plate, done at PND70. The next day, marble burying test and forced swim test were done with the interval of 60 min. One to two hours after the last behavioral test, rats were euthanized for tissue collection.For all procedures requiring euthanasia, rats were placed in a CO₂ chamber and exposed to gradually increasing carbon dioxide at a displacement rate of 20–30% of the chamber volume per minute to ensure smooth induction of anesthesia and loss of consciousness. Once deep anesthesia was confirmed by the absence of reflexes, decapitation was performed for rapid tissue collection. This procedure was conducted in accordance with standard humane guidelines and institutional animal welfare requirements.

Once deep anesthesia was confirmed by the absence of reflexes, CO₂ exposure was continued until euthanasia was completed. This method is considered humane and is consistent with institutional and international animal welfare standards. Behavioral tests were conducted using a fixed sequence progressing from less to more stressful paradigms, including the open field test, hot plate test, marble burying test, and forced swim test. This order was selected to minimize carry-over effects between tests, with the forced swim test performed last due to its high emotional and physiological load. A minimum inter-test interval of 60 min was maintained, and all experimental groups were exposed to the same testing order to reduce procedural variability^46,47^.

### Social isolation

The isolation protocol was started since the rats were at the age of PND20 (long-term social isolation) or PND45 (short-term social isolation). Social isolation involved depriving the rat of contact and coexistence with other rats. Each rat was placed alone in a cage for a specified period of time, with free access to water and food^8^. All the tests were done at the age of PND70.

### Open field test

The open field test was performed in a transparent Perspex arena (30 × 30 × 40 cm) divided into 16 equal squares (Tajhiz-Gostar Omid Iranian Co, Tehran, Iran). During the 5-min session, crossings, time spent in the central squares, rearing, grooming, sniffing, and climbing were recorded. In this test, we recorded locomotor activity, time spent in the middle squares, sniffing, grooming, and rearing. It should be noted that the dimensions of the open field apparatus used in this study are adequate primarily for the assessment of locomotor activity and stereotyped behaviors rather than a comprehensive anxiety test. Therefore, anxiety-like behavior was interpreted cautiously and indexed only by time spent in the central squares, as a complementary parameter. A crossing was counted when all four paws of the rat entered a new square^48^. Sniffing was considered stereotyped when it occurred repeatedly without orientation toward novel stimuli, distinguishing it from exploratory sniffing^49^. Anxiety-like behavior was assessed using time spent in the central area and rearing frequency as proxy indices. Although the elevated plus maze is a well-established paradigm for anxiety assessment, it was not used in the present study in order to limit cumulative stress exposure across repeated behavioral testing^50^. Locomotor activity was evaluated by recording the number of all crossings of each rat through the arena. Time spent in the four middle squares was recorded to assess anxiety-like behavior^51,52^. Rearing behavior is considered a complex and context-dependent behavioral phenotype that may reflect exploratory drive, emotional arousal, or stress coping strategies rather than anxiety alone. Previous evidence suggests that increased rearing under aversive or novel conditions may reflect anxiolytic responses associated with enhanced exploratory behavior, whereas reductions in rearing may indicate either decreased exploration or heightened emotional suppression. Accordingly, alterations in rearing behavior in the present study were interpreted cautiously and in conjunction with other behavioral measures rather than as a direct index of anxiety alone^50,53^. Stereotypic behaviors (grooming and sniffing) were also recorded. Stereotyped behavior is a behavioral pattern in which various motor activities are accompanied by repetition and intensity^54–56^.

### Hot plate

Thermal nociception was assessed using a hot plate set at 50 °C, and latency to paw licking or jumping was recorded, with a 100-s cutoff (Tajhiz-Gostar Omid Iranian Co, Tehran, Iran). In order to determine pain threshold, the rat was placed on the hot plate, and the exact time spent (seconds) to the rats started to lick their paws was recorded. If the rat does not feel pain within 100 s, the test is stopped and the result of the 100-second is announced^57,58^.

### Marble burying test

Marble burying test measures obsessive-compulsive disorder (OCD)-like behavior in rodents. OCD-like behavior in this behavioral test was determined via using standard glass toy marbles. All these marbles had the same color and style (15 mm diameter, 5–6 g in weight). In this test, the marbles were placed on the surface of the bedding in 2 rows of 5 marbles (10 marbles). OCD-like behavior was determined via recording the number of buried marbles during 30 min^59^.

### Forced swim test

Forced swim test was a cylindrical transparent container filled with water (20–22 °C) up to 2/3 of it. The forced swim test was conducted in a cylindrical container (height: 50 cm, diameter: 20 cm), filled with water to a depth of 30 cm^60^. The duration of the test was 300 s, and the rat was placed inside the container and had to swim to avoid drowning. The time spent with immobility or climbing (seconds) were recorded. Immobility has been considered as depressive-like state^61^. No habituation session was conducted prior to the forced swim test. A single-session paradigm was employed, and immobility and climbing durations were quantified as primary behavioral outcomes. Latency to immobility and swimming duration were not analyzed. Trained evaluators were physically present during each session and recorded behavioral endpoints immediately according to predefined, standardized scoring criteria.

### Western blotting

Western blot analyses were performed as previously described with some modifications [Babaei et al., 2018; Jabarpour et al., 2018]. After completion of behavioral testing, rats were euthanized as described above, and the brains were rapidly removed for tissue dissection.

Brains were rapidly removed and sectioned using a rat brain matrix. Bilateral medial prefrontal cortex tissue was dissected according to the Paxinos and Watson rat brain atlas, corresponding approximately to + 4.2 to + 2.2 mm relative to bregma^62^. For western blotting, tissue was lysed with ripa buffer. The lysates were removed by centrifugation at 14,000 rpm for 20 min at 4 °C. Protein concentration was determined by the Bradford Protein Quantification kit (DB0017, DNAbioTech, Iran) according to manufacturer’s instructions. The tissue lysates were mixed with equal volume of 2X Laemmli sample buffer. Lysates (20 µg) were then subjected to SDS-PAGE after a 5 min boiling and subsequently transferred to a 0.2 μm immune-Blot™ polyvinylidene difluoride (PVDF) membrane (Cat No: 162–017777; Bio-Rad Laboratories, CA, USA). The membranes were then blocked with 5% BSA (Cat No: A-7888; Sigma Aldrich, MO, USA) in 0.1% Tween 20 for 1 h. Then, the membranes were incubated with anti-DAT and anti-β actin-loading control antibodies (1/2500, Cat No: ab8227, Abcam) for 1 h at room temperature. Subsequently, membranes were washed thrice with TBST, and incubated with goat anti-rabbit IgG H&L (HRP) (1/10000, Cat No: ab6721; Abcam) secondary antibody. The membranes were then incubated with enhanced chemiluminescence (ECL) for 1–2 min. Protein expression was normalized to β-actin. Densitometry of protein bands was performed using the gel analyzer Version 2010a software (NIH, USA), such that, the percentage area under the curve of each band was divided by the percentage area under the curve of its corresponding actin band, and then calculated values were compared between groups as we described previously. All western blot images were reviewed in accordance with the journal’s digital image integrity policy. Full-length and uncropped blot images corresponding to Fig. 6 are provided in the Supplementary Information.

### Statistical analyses

The present study performed data analyses using SPSS software. Normality of data distribution was assessed using the Shapiro–Wilk test prior to performing factorial ANOVA. In order to determine the potential significant effects of all variable (social isolation, sex, and social isolation*sex). Data were analyzed using two-way ANOVA with sex (male, female) and isolation condition (control, short-term isolation, long-term isolation) as between-subject factors, followed by Tukey’s post hoc test where appropriate. Also, post hoc Tukey test was used to compare the results between groups. Data was expressed as mean ± SD and *P* < 0 0.05 was the level of statistical significance.

## Results

### Locomotor activity and anxiety-like behavior

**Fig. 1:**
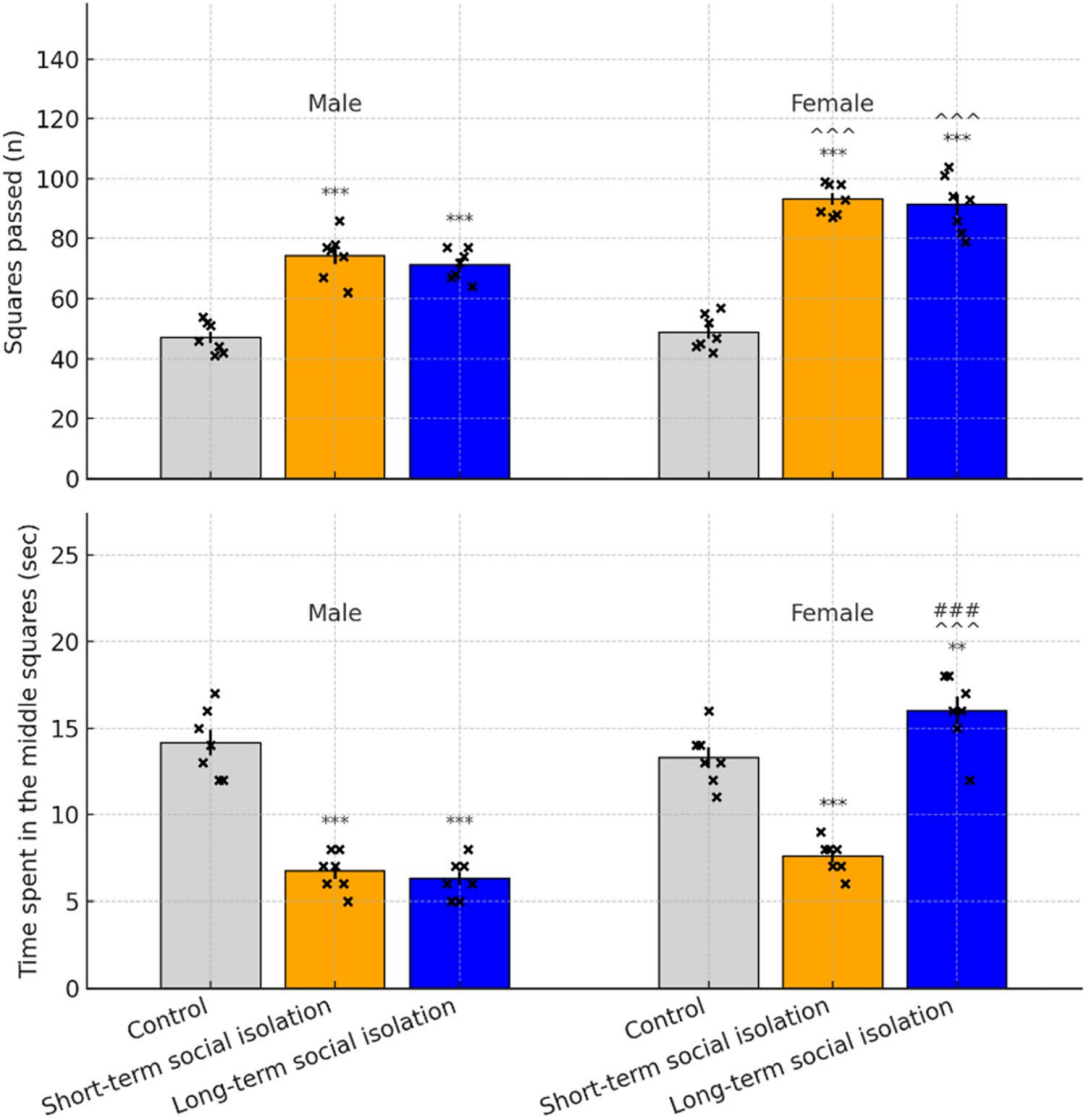
Squares passed (locomotor activity) and time spent in the middle squares (anxiety-like behavior) in both sexes in control, and short- (25 days) and long- (50 days) term social isolation groups [***P < 0.001 and **P < 0.01 compared with related control; ^^^*P* < 0.001 compared with respective male group; ###*P* < 0.001 compared with related short-term social isolation; *n* = 7]. Individual data points are shown overlaid on mean ± SD bars.

*Locomotor activity-* Two-way ANOVA reports were as follows: The effect of social isolation (F_2, 36_ = 127.12, *P* < 0.001), sex (F_1, 36_ = 43.79, *P* < 0.001), and social isolation*sex (F_2, 36_ = 8.37, *P* < 0.001). Two-way ANOVA revealed significant main effects of isolation condition (F2, 36 = 127.12, *P* < 0.001), sex (F1,36 = 43.79, *P* < 0.001), and isolation condition × sex interaction (F2,36 = 8.37, *P* < 0.001). Post hoc Tukey comparisons showed that locomotor activity was significantly higher in both short-term and long-term socially isolated rats of both sexes than in their respective controls (all *P* < 0.001). Female rats showed greater locomotor activity than the corresponding male groups (*P* < 0.001) (Fig. 1).

*Anxiety-like behavior-* Two-way ANOVA reports were as follows: The effect of social isolation (F_2, 36_ = 65.13, *P* < 0.001), sex (F_1, 36_ = 46.71, *P* < 0.001), and social isolation*sex (F_2, 36_ = 47.80, *P* < 0.001). Post hoc Tukey test showed that anxiety-like behavior was significantly increased in socially isolated male rats (both short- and long-term protocol) (*P* < 0.001). While, in females, only short-term social isolation led to increased anxiety-like behavior (*P* < 0.001). However, long-term social isolation in females showed a decreased anxiety-like behavior than controls (*P* < 0.01), respective males (*P* < 0.001), and short-term social isolation (*P* < 0.001) (Fig. 1).

### Grooming, sniffing, and rearing

**Fig. 2:**
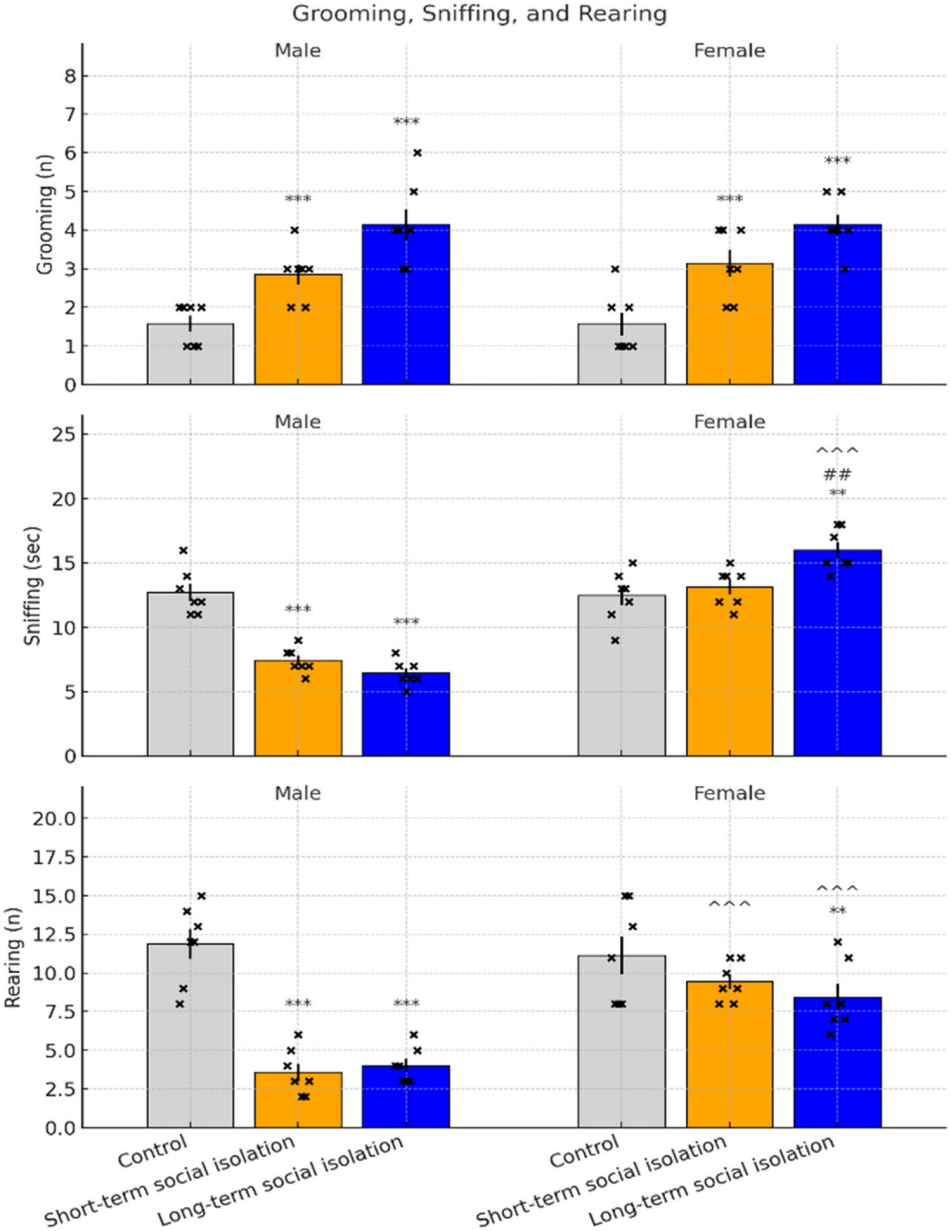
Grooming, sniffing, and rearing in both sexes in control, and short- (25 days) and long- (50 days) term social isolation groups [***P < 0.001 and **P < 0.01 compared with related control; ^^^*P* < 0.001 compared with respective male group; ##*P* < 0.01 compared with related short-term social isolation; *n* = 7]. Individual data points are shown overlaid on mean ± SD bars.

*Grooming-* Two-way ANOVA reports were as follows: The effect of social isolation (F_2, 36_ = 36.60, *P* < 0.001), sex (F_1, 36_ = 0.15, *P* > 0.05), and social isolation*sex (F_2, 36_ = 0.15, *P* > 0.05). Post hoc Tukey test showed that grooming was significantly increased in both sexes of socially isolated rats (both short- and long-term protocol) (*P* < 0.001) (Fig. 2).

*Sniffing-* Two-way ANOVA reports were as follows: The effect of social isolation (F_2, 36_ = 7.98, *P* < 0.001), sex (F_1, 36_ = 113.27, *P* < 0.001), and social isolation*sex (F_2, 36_ = 37.26, *P* < 0.001). Post hoc Tukey test showed that sniffing was significantly decreased in socially isolated male rats (both short- and long-term protocol) (*P* < 0.001). While, in females, only long-term social isolation led to increased sniffing compared with controls (*P* < 0.01), respective males (*P* < 0.001), and short-term social isolation (*P* < 0.01) (Fig. 2).

*Rearing-* Two-way ANOVA reports were as follows: The effect of social isolation (F_2, 36_ = 27.35, *P* < 0.001), sex (F_1, 36_ = 23.63, *P* < 0.001), and social isolation*sex (F_2, 36_ = 9.24, *P* < 0.001). Post hoc Tukey test showed that rearing was significantly decreased in socially isolated male rats (both short- and long-term protocol) (*P* < 0.001). While, in females, only long-term social isolation led to decreased rearing compared with controls (*P* < 0.01). Rearing in socially isolated females was more than respective males (*P* < 0.001) (Fig. 2).

### Pain threshold

**Fig. 3:**
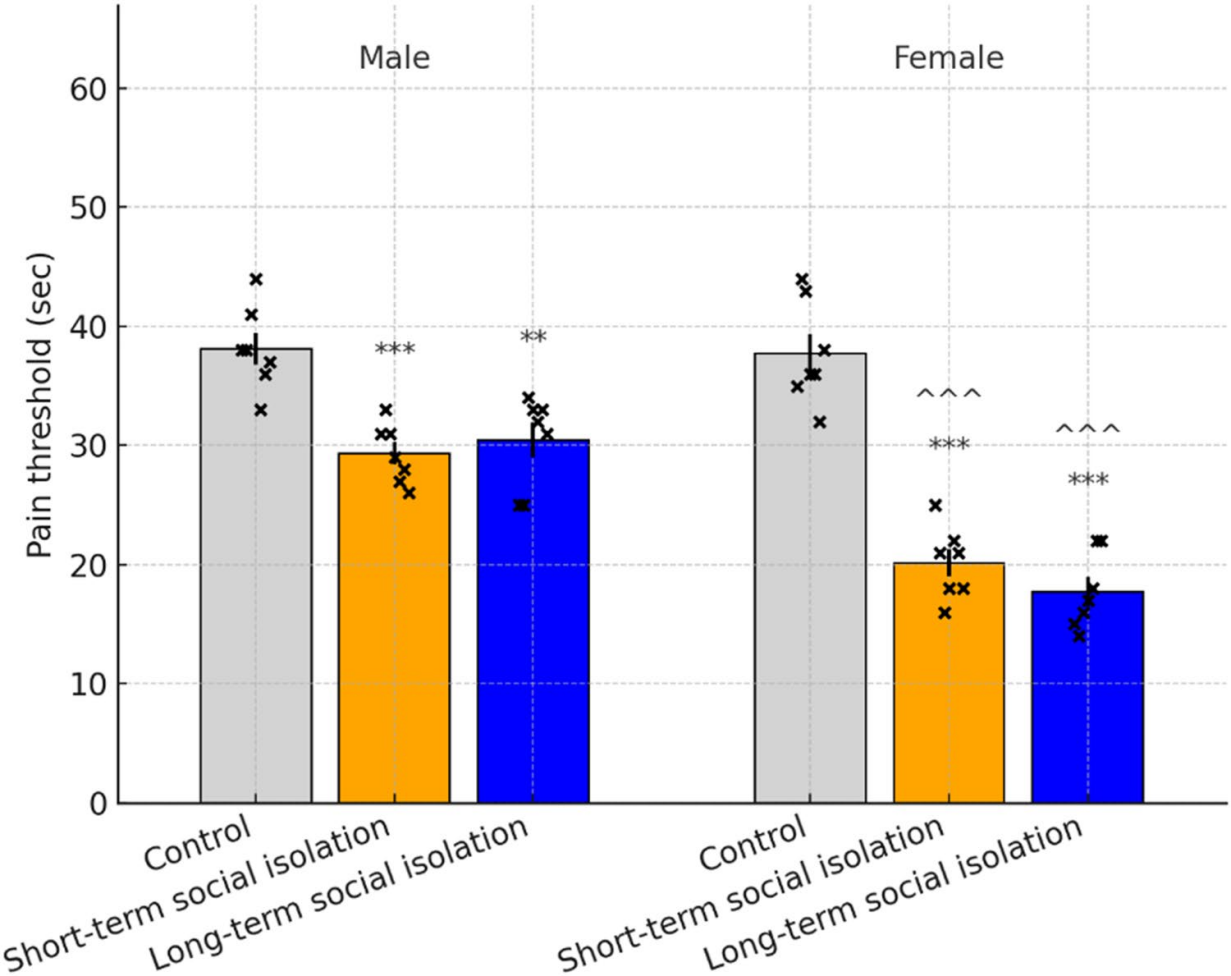
Pain threshold in both sexes in control, and short- (25 days) and long- (50 days) term social isolation groups [***P < 0.001 and **P < 0.01 compared with related control; ^^^*P* < 0.001 compared with respective male group; *n* = 7]. Individual data points are shown overlaid on mean ± SD bars.

Two-way ANOVA reports were as follows: The effect of social isolation (F_2, 36_ = 71.75, *P* < 0.001), sex (F_1, 36_ = 48.54, *P* < 0.001), and social isolation*sex (F_2, 36_ = 11.71, *P* < 0.001). Post hoc Tukey test showed that pain threshold was significantly decreased in socially isolated male rats (short-term: *P* < 0.001, long-term: *P* < 0.01). In females, both protocols of social isolation led to decreased pain threshold (*P* < 0.001). Pain threshold in socially isolated females was lesser than respective males (*P* < 0.001) (Fig. 3).

### Marbles buried

**Fig. 4:**
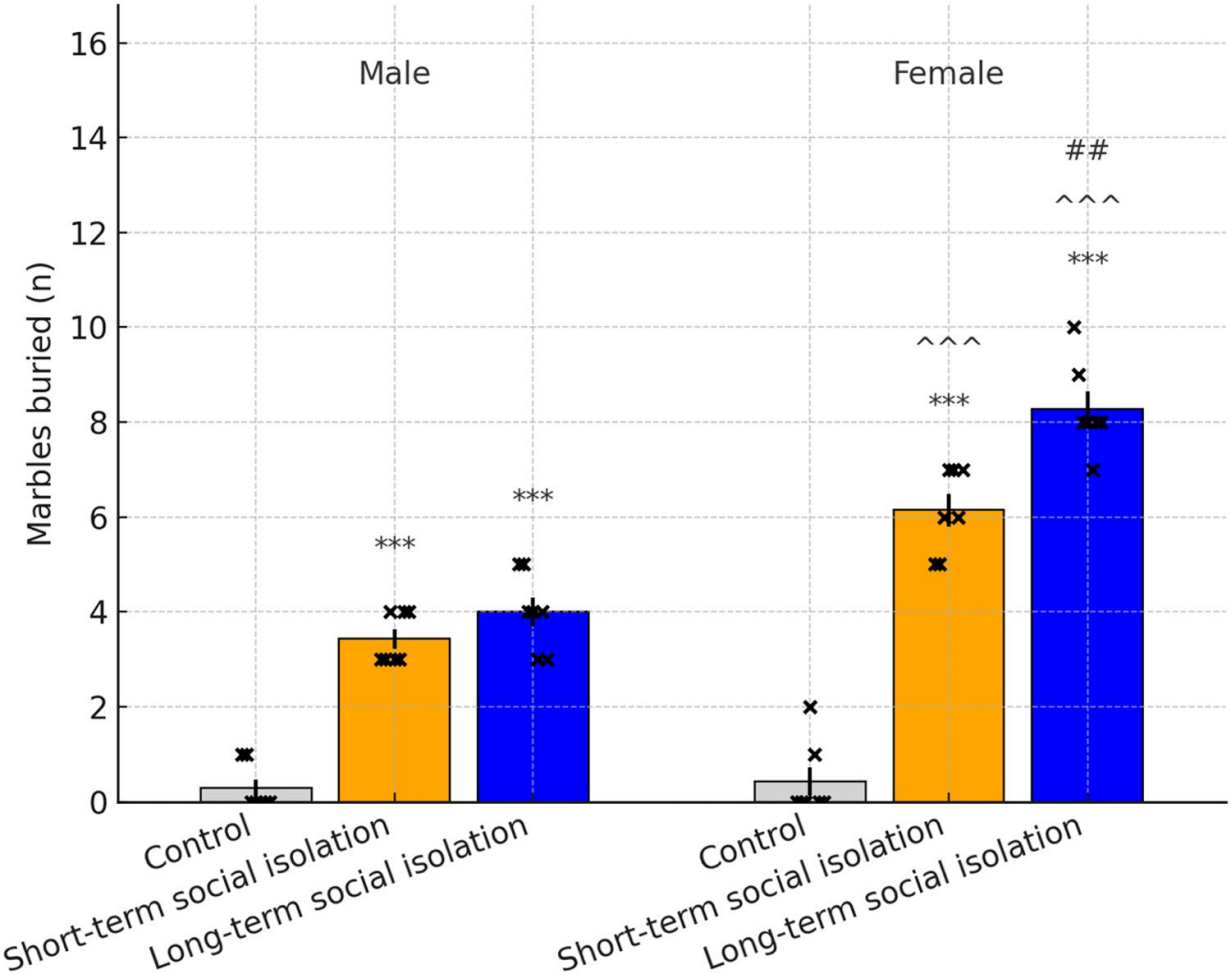
Marbles buried (OCD-like behavior) in both sexes in control, and short- (25 days) and long- (50 days) term social isolation groups [***P < 0.001 compared with related control; ^^^*P* < 0.001 compared with respective male group; ##*P* < 0.01 compared with related short-term social isolation; *n* = 7]. Individual data points are shown overlaid on mean ± SD bars.

Two-way ANOVA reports were as follows: The effect of social isolation (F_2, 36_ = 218.23, *P* < 0.001), sex (F_1, 36_ = 101.35, *P* < 0.001), and social isolation*sex (F_2, 36_ = 26.07, *P* < 0.001). Post hoc Tukey test showed that marble buried was significantly increased in socially isolated male and female rats (*P* < 0.001). Females in both social isolation groups buried more marbles than males (*P* < 0.001). Also, females exposed to long-term social isolation buried more marbles than females exposed to short-term social isolation (*P* < 0.01) (Fig. 4).

### Immobility and climbing

**Fig. 5:**
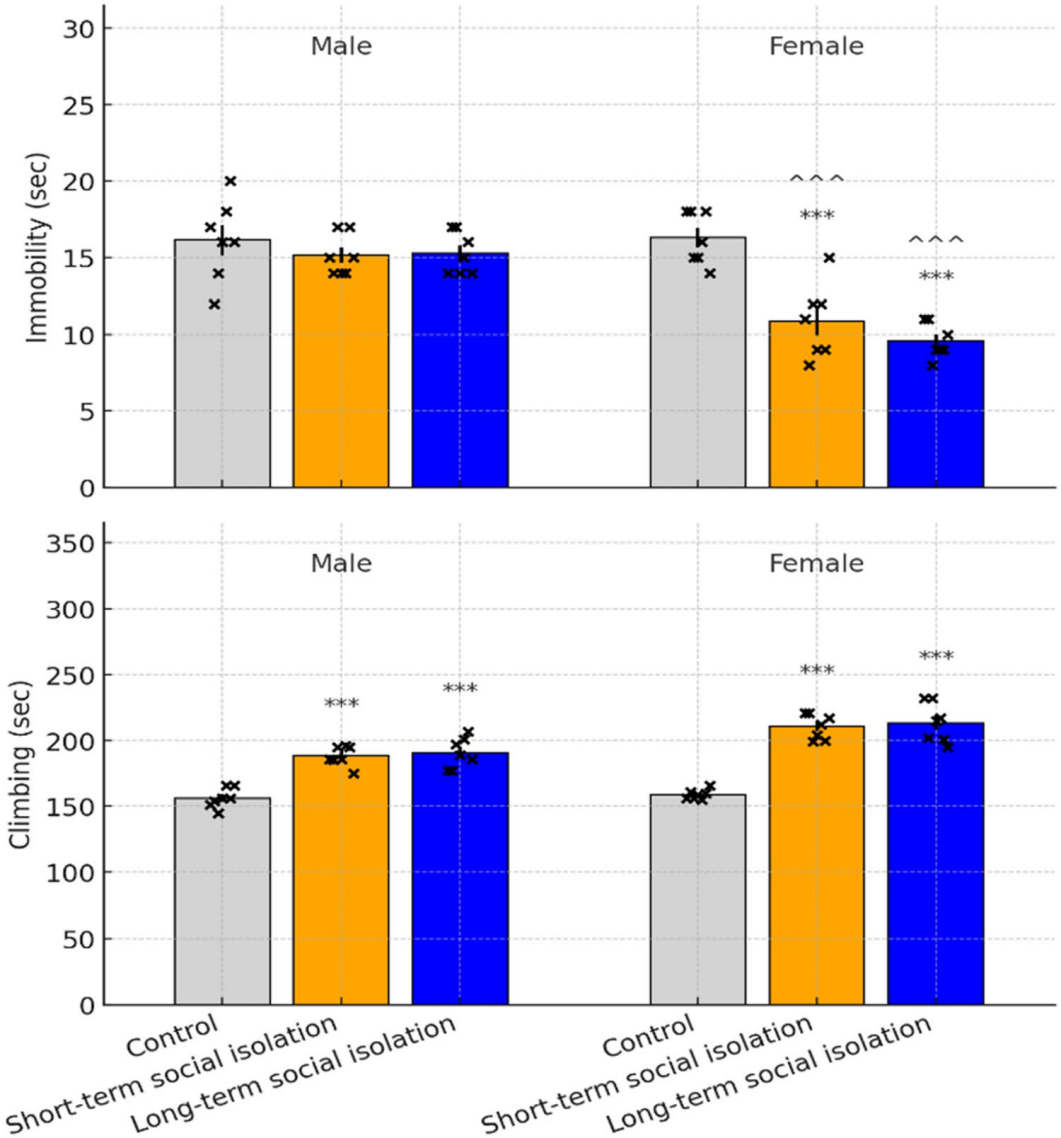
Immobility (depressive-like behavior) and climbing in both sexes in control, and short- (25 days) and long- (50 days) term social isolation groups [***P < 0.001 compared with related control; ^^^*P* < 0.001 compared with respective male group; *n* = 7]. Individual data points are shown overlaid on mean ± SD bars.

*Immobility-* Two-way ANOVA reports were as follows: The effect of social isolation (F_2, 36_ = 17.05, *P* < 0.001), sex (F_1, 36_ = 33.14, *P* < 0.001), and social isolation*sex (F_2, 36_ = 9.54, *P* < 0.001). Post hoc Tukey test showed that immobility was only decreased in females (compared with control and respective males: *P* < 0.001) (Fig. 5).

*Climbing-* Two-way ANOVA reports were as follows: The effect of social isolation (F_2, 36_ = 89.90, *P* < 0.001), sex (F_1, 36_ = 27.67, *P* < 0.001), and social isolation*sex (F_2, 36_ = 4.77, *P* < 0.05). Post hoc Tukey test showed that climbing was significantly increased in all socially isolated rats (*P* < 0.001) (Fig. 5).

### DAT protein levels in the prefrontal cortex

**Fig. 6:**
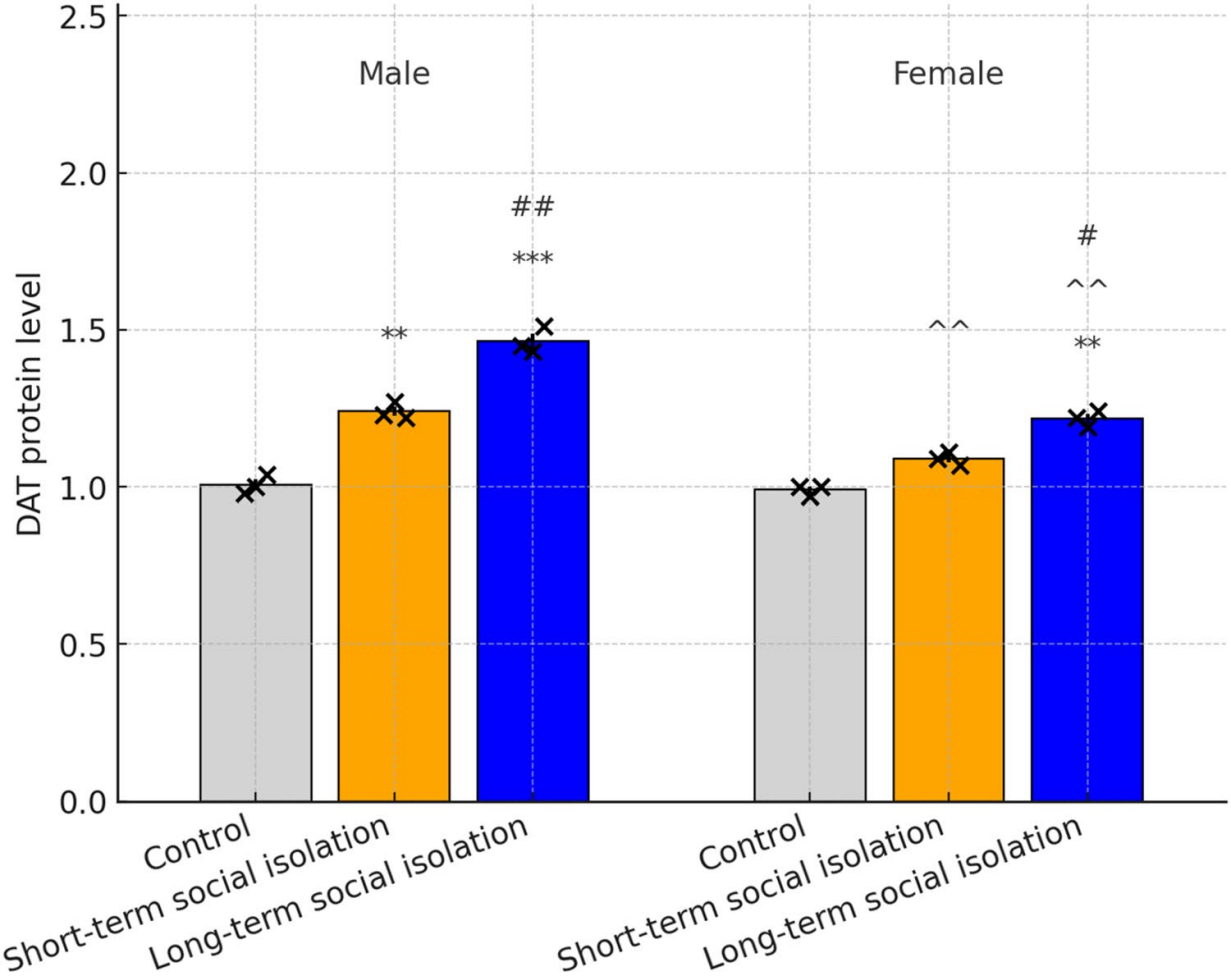
DAT protein levels in the medial prefrontal cortex in both sexes in control, and short- (25 days) and long- (50 days) term social isolation groups [***P < 0.001 and **P < 0.01 compared with related control; ^^*P* < 0.01 compared with respective male group; ##*P* < 0.01 and #*P* < 0.05 compared with related short-term social isolation; *n* = 3]. For western blot analysis, three representative samples per group were used, a common practice in protein expression studies to minimize animal use while ensuring reproducibility. Upper panels represent representative DAT and β-actin bands; lower panels show quantitative densitometric analysis normalized to β-actin. Individual data points are shown overlaid on mean ± SD bars. Full-length, uncropped western blot images are provided in the Supplementary Information in accordance with journal guidelines.

Two-way ANOVA reports were as follows: The effect of social isolation (F_2, 12_ = 223.58, *P* < 0.001), sex (F_1, 12_ = 109.05, *P* < 0.001), and social isolation*sex (F_2, 12_ = 25.54, *P* < 0.001). Post hoc Tukey test showed that DAT protein levels were increased in short-term (*P* < 0.01) and long-term (*P* < 0.001) socially isolated male rats. However, only long-term social isolation increased DAT protein levels in females (*P* < 0.01). DAT protein levels were lower in both groups of socially isolated female rats than males (*P* < 0.01). Also, long-term social isolation in males (*P* < 0.01) and in females (*P* < 0.05) showed higher DAT levels than respective short-term rats (Fig. 6).

## Discussion

Unlike many previous studies that applied a single social isolation protocol, the present study directly compared short- and long-term isolation initiated at distinct developmental time points. Our findings demonstrate that initiation of isolation during earlier neurodevelopment (PND20) produced more robust and sex-specific alterations in behavior and DAT expression than isolation beginning at PND45. These findings align with evidence indicating heightened vulnerability of the mPFC to stressors during early adolescence and support the notion that timing of isolation may outweigh duration alone in shaping affective outcomes^63^. The results of the present research revealed significant sex differences between short- and long-term social isolation in behavioral functions and DAT protein levels. It was shown that locomotor activity was increased in both protocols of social isolation, while females showed a more hyperlocomotion. Anxiety-like behavior was increased (decreased the time spent in the middle squares) in both protocols of social isolation in males, while in females, only short-term social isolation showed this effect, and interestingly, long-term social isolation increased the time spent in the middle squares. Grooming was increased in both protocols of social isolation with no sex differences. Sniffing duration was decreased in both protocols of social isolation in males, while in females, only long-term social isolation increased it. Rearing was decreased in both protocols of social isolation in males, while in females, only long-term social isolation decreased it. Pain threshold was decreased in both protocols of social isolation in males, while this effect was more significant in females. Marbles buried (OCD-like behavior) was increased in both protocols of social isolation in males, while this effect was more significant in females. Immobility was only decreased in both protocols of social isolation in females. Climbing was increased in both protocols of social isolation with no sex differences. Eventually, DAT levels were increased in both protocols of social isolation in males, while this effect was only observed in long-term socially isolated female rats.

The present findings show that social isolation was accompanied by distinct behavioral alterations in males and females, together with sex- and protocol-dependent changes in DAT expression within the medial prefrontal cortex. In socially isolated males, increased DAT protein levels were observed in parallel with marked changes in locomotor, compulsive-like, and center-related open-field behaviors. In females, DAT upregulation was evident only after the longer isolation protocol, suggesting that the molecular response may differ according to sex and exposure schedule. However, because DAT function was not directly manipulated in the present study, these findings should be interpreted as associative rather than causal. Further studies using pharmacological or genetic approaches are needed to determine whether altered DAT signaling directly contributes to the behavioral phenotype observed after social isolation^38,39,44^.

Due to the design of the behavioral tests, locomotor activity changes can affect the results of anxiety- and depressive-like behaviors. Hyperlocomotion may lead to more locomotor activity in the open field and forced swim test, interpreting as an antidepressant or anti-anxiety effect. Increased locomotor activity has been shown in socially isolated animals. Previous study has shown that social isolation started from adolescence leads to significant hyperlocomotion in rats^8^. Elevated locomotion in the open field test and increased closed-arm entries on the elevated plus maze have been observed in rats exposed to adolescent social isolation^64^. Additionally, socially isolated female Long Evans rats show elevated locomotor activity in the open field test^65^. Although some inconsistent reports have been shown^66,67^. Therefore, altered locomotion induced by social isolation may change the rats’ behavioral results in the open field and forced swim tests. The present study also showed increased locomotor activity in both sexes of socially isolated rats, while immobility (depressive-like behavior) was unaffected in male and decreased in females, and climbing was increased in both sexes. These effects may be related to elevated locomotor activity. Although anxiety-like behavior was increased (time spent in the middle squares was decreased) in males and short-term socially isolated females, while long-term socially isolated females showed an opposite behavior. Rearing behavior was also decreased in all groups. As mentioned, rearing behavior has been considered as both anxiolytic and anxiogenic behavioral phenotype^50,68,69^. In the present research, the results of anxiety-like behaviors were inconsistent. We showed two inconsistent behaviors of socially isolated female rats in the open field test following two protocols of social isolation, suggesting the role of the duration of social isolation.

We also showed OCD-like behavior in both sexes of socially isolated rats. Previous study has shown that social isolation significantly leads to OCD-like behavior in the marble burying test in rats^8^. Other study has shown that early social isolation significantly induces OCD-like behaviors in rats^70^. Furthermore, a related study has shown increased marbles buried in rats exposed to chronic social isolation^71^. In line with previous findings, we showed that social isolation may lead to significant OCD-like behavior, and higher level of this behavioral phenotype was shown in females exposed to long-term social isolation. The results also showed increased grooming in both sexes and sniffing only in females, while sniffing was decreased in males. As mentioned, both grooming and sniffing are considered as stereotyped behaviors. It has been noted that social isolation leads to decreased stereotyped behaviors in socially isolated rats^72^. Other study has shown increased grooming in male rats experiencing social isolation from PND21-70^73^. Increased aggressive grooming has also been reported in rats experiencing social isolation^74,75^. It has also been revealed that social isolation leads to increased sniffing and grooming in both sexes of the offspring exposed to early life overnutrition^76^. Additionally, social isolation rearing leads to increased self-grooming and locomotor activity, and decreased anogenital sniffing in rats^77^. It has also been demonstrated that social isolation gradually increases sniffing depends on the isolation period. While, in aged rats, more prolonged social isolation (4 weeks) alleviates the 2-week social isolation-induced increased sniffing behavior^78^. Furthermore, 5-week social isolation in male mice at adulthood increases sniffing behavior in the resident-intruder test^79^. The present study, for the first time, showed sex differences in sniffing behavior in the open field test in socially isolated rats.

Our data showed that DAT protein levels were increased in socially isolated males, while in females, only long-term social isolation increased DAT levels. Also, both groups of socially isolated males had more DAT levels than females. DAT (also named as SLC6A3) is a dopamine transporter that is implicated in dopamine releasing and reuptaking from the synapse and extrasynaptic space into neurons, leading to the induction of significant effects on dopaminergic signaling^80^. DAT is a major regulator of extracellular dopamine availability and has been implicated in several psychiatric and stress-related phenotypes. Previous studies have linked altered DAT function to anxiety-like behavior, locomotor changes, compulsive-like behavior, and stress vulnerability. For instance, it has been shown that DAT inhibitors meliorate cognitive impairments in Alzheimer’s disease^81^. Previous studies have found a significant relationship between DAT dysfunction and posttraumatic stress disorder (PTSD)^82,83^. An increase in horizontal activity and stereotyped head-weave activity have been reported in DAT knock-out female rats^84^. Other study has reported that anxiety-like behavior and locomotor activity are highly increased in both sexes of DAT knock-out rats^37^. Neonatal maternal separation leads to anxiety-like behavior in rats via increasing DAT levels in the medial prefrontal cortex^85^. Previous study has suggested that the improvement effects of propofol on anxiety level in sleep-deprived DAT knock-out rats may be related to the function of DAT in the ventral tegmental area (VTA)^86^. Ruzu herbal bitters (RHB, with possible neurotoxic effects) has been shown to induce anxiety-like behavior and impairs locomotor activity in the open field test via increasing both DAT and serotonin transporter (SERT)^87^. Furthermore, DAT levels are increased in a muscleblind-like 2 (Mbnl2) knock-out mice (a model of CNS alterations), leading to depressive-like behaviors and cognitive deficits^88^. Of note, another related study has demonstrated that DAT knock-out rats exhibit increased locomotor activity and a transient anxiety profile, along with stereotypy and OCD-like behaviors in the marble burying test^89^. Although excessive grooming has been associated with compulsive-like behavior, in the present study grooming was categorized as a stereotyped behavior rather than a direct OCD-like index. Marble burying was used as the primary and validated measure of OCD-like behavior^90^. DAT heterozygous rats (born from DAT knock-out father) also show increased OCD-like behavior in the marble burying test^91^. It has been shown that lithium treatment significantly reduces high protein levels of DAT in rats exposed to chronic stress, leading to decreased anxiety^92^. Rats experiencing unavoidable stress exhibit a decreased dopaminergic output in the nucleus accumbens shell along with decreases of DAT binding sites^93^. In addition, DAT knock-out mice show baseline reductions in immobility (depressive-like) behavior and grooming time in the splash test of grooming behavior^94^. It has also been shown that repeated restraint stress significantly increases DAT activity in the striatum of mice^95^. Importantly, a previous study has shown that dopamine release and uptake, and DAT levels are increased in the nucleus accumbens of socially isolated rats^39^. Correlation analyses between behavioral measures and DAT protein levels were not performed due to the limited sample size used for molecular analysis, which would have resulted in insufficient statistical power and potentially unreliable interpretations. However, evidence on DAT changes in socially isolated animals is so limited. Therefore, previous findings have shown the important role of DAT function in mediating anxiety-, depressive-, and OCD-like behaviors, and locomotor activity. Also, previous reports have shown the significant impact of stress (including social isolation) on DAT functions, resulting in behavioral alterations. For the first time, the present study showed that social isolation leads to increased DAT protein levels in the prefrontal cortex in males, while only long-term social isolation showed this effect in females.

Several limitations should be acknowledged. First, estrous cycle stages were not monitored in female rats, which may have contributed to variability in behavioral responses^43^. Second, the western blot analysis was performed on a limited number of samples per group, which reduces the strength of molecular inference and precluded robust correlation analyses between DAT levels and behavioral measures. Third, DAT protein expression was assessed, but functional assays of dopamine uptake or direct manipulations of DAT were not performed; therefore, mechanistic conclusions should be avoided. Fourth, some behavioral outcomes, particularly center-related open-field measures and forced swim parameters, may have been influenced by changes in locomotor activity, and thus should be interpreted cautiously. Finally, the two isolation protocols differed not only in duration but also in developmental onset (PND20 vs. PND45), meaning that the present design cannot fully disentangle the effects of isolation duration from the effects of exposure timing during neurodevelopment^38,42^.

## Conclusion

In conclusion, the present findings indicate that short- and long-term social isolation are associated with sex-dependent alterations in locomotor activity, stereotyped behaviors, nociceptive threshold, compulsive-like behavior, and selected stress-coping behaviors in rats. The behavioral response related to time spent in the central area of the open field appeared to differ between female isolation protocols and should therefore be interpreted with caution. In parallel, social isolation was associated with increased DAT protein levels in the medial prefrontal cortex in males under both isolation paradigms, whereas in females this increase was observed only after long-term isolation. Taken together, these findings suggest that the behavioral consequences of social isolation may be accompanied by sex- and duration-dependent alterations in prefrontal DAT expression. However, further studies are required to determine whether DAT changes play a causal role in these behavioral outcomes.

### ***Supplementary Information***

All full-length and uncropped western blot images corresponding to Fig. 6 are provided in the Supplementary Information file. Multiple exposures were acquired where necessary to ensure optimal signal detection without saturation, and representative exposures are presented. No selective or non-linear image manipulation was applied. Only uniform adjustments of brightness and contrast were performed across the entire image when necessary for clarity. All delineations between gel regions are clearly indicated. These materials are provided to ensure transparency and compliance with the journal’s digital image integrity requirements.

## Supplementary Information

Below is the link to the electronic supplementary material.

Supplementary Material 1

## Data Availability

The datasets generated and/or analyzed during the current study are available from the corresponding author upon reasonable request.
