# Supplementary material for: Sex-dependent effects of short- and long-term social isolation on behavior and medial prefrontal DAT in rats
Source: Sci Rep. 2026 Apr 27;16:19712. doi: 10.1038/s41598-026-48488-1 (PMC13316144; doi:10.1038/s41598-026-48488-1)

Supplementary Information

Full-length, uncropped western blot images corresponding to Figure 6 are provided below. All blots were processed and imaged under identical experimental conditions. Representative exposures (3 seconds) are shown, selected based on optimal signal detection without saturation. No non-linear image manipulation was performed. Only uniform brightness and contrast adjustments were applied across the entire image when necessary. Molecular weight markers are indicated. Expected band sizes: DAT (~50 kDa) and β-actin (~43 kDa). Any cropping applied in the main manuscript figures was performed solely for presentation clarity and did not exclude any relevant bands.

Figure S1. DAT (50 kDa) – P1 exposure (3 s)

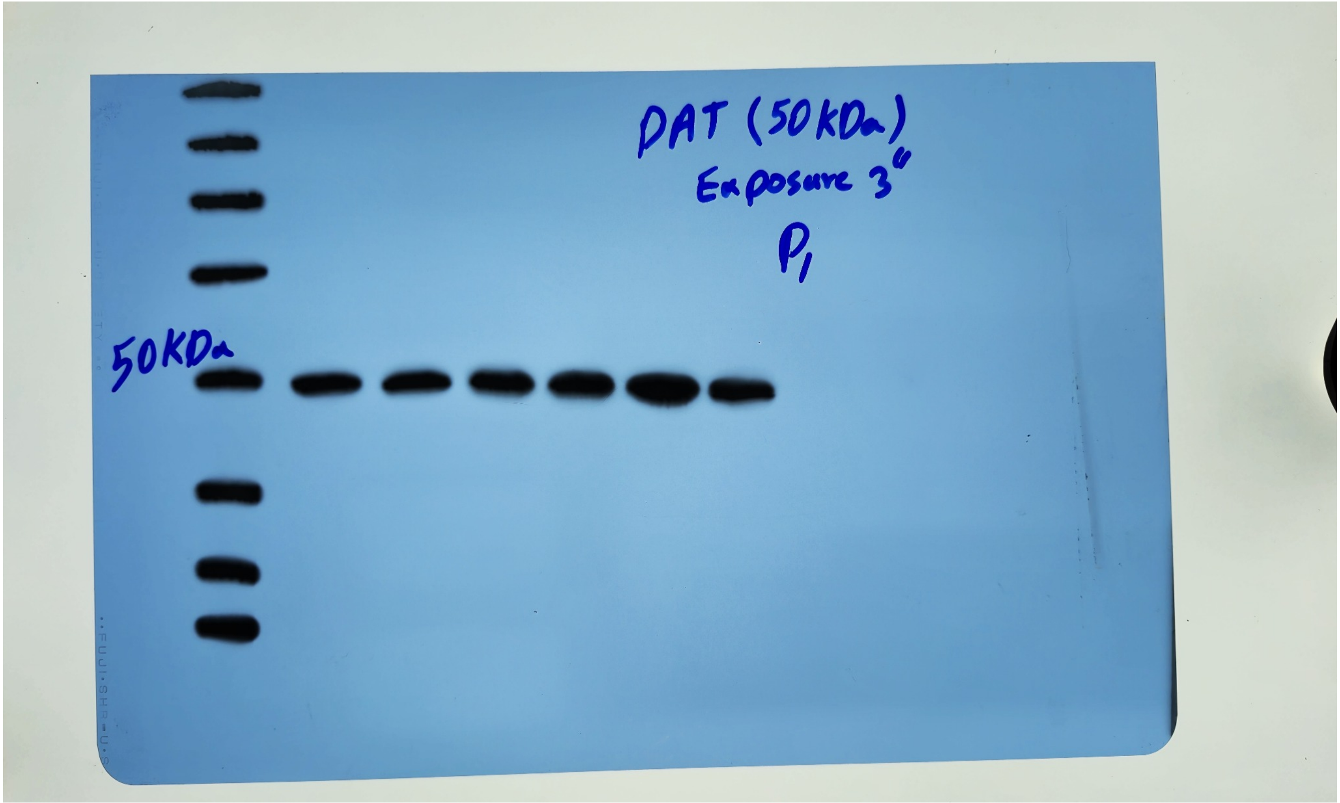

Figure S2. DAT (50 kDa) – P2 exposure (3 s)

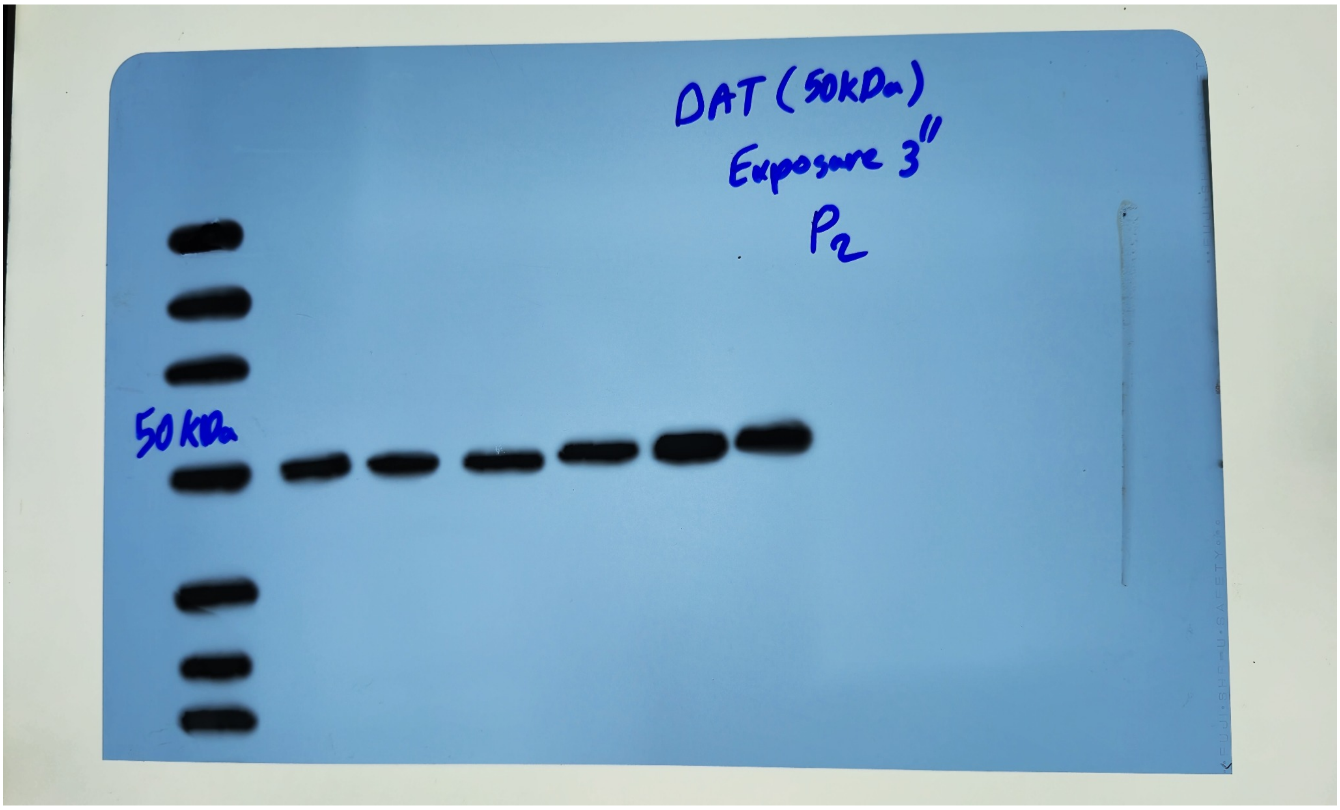

Figure S3. DAT (50 kDa) – P3 exposure (3 s)

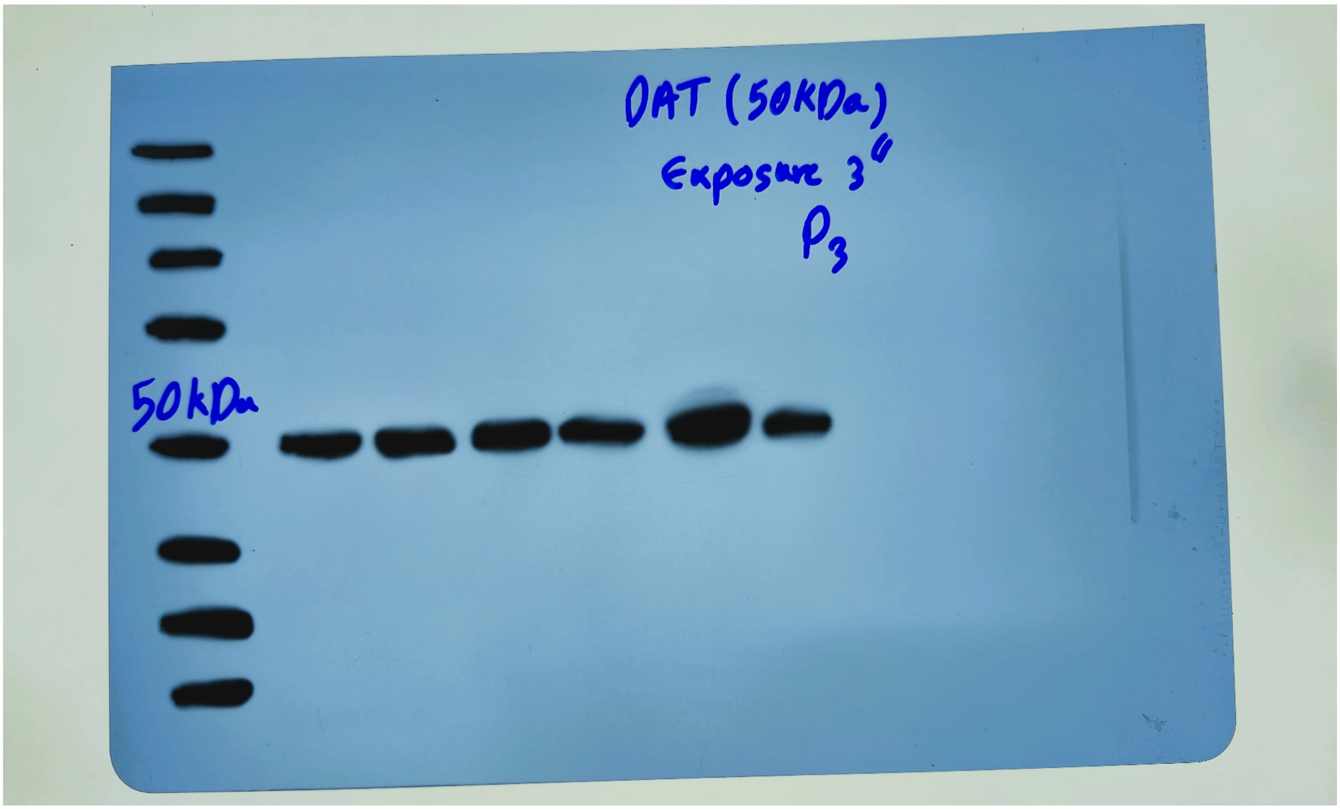

Figure S4. β-actin (43 kDa) – P1 exposure (3 s)

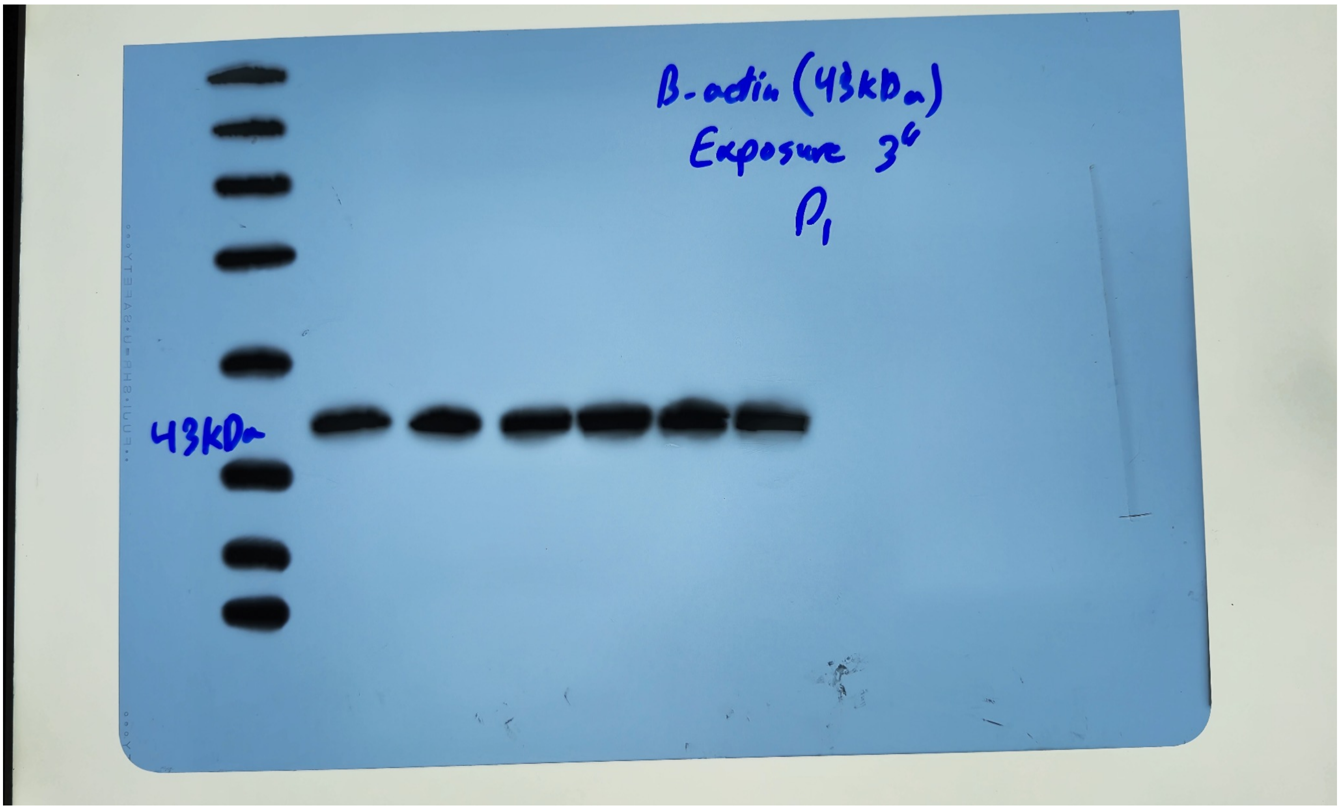

Figure S5. β-actin (43 kDa) – P2 exposure (3 s)

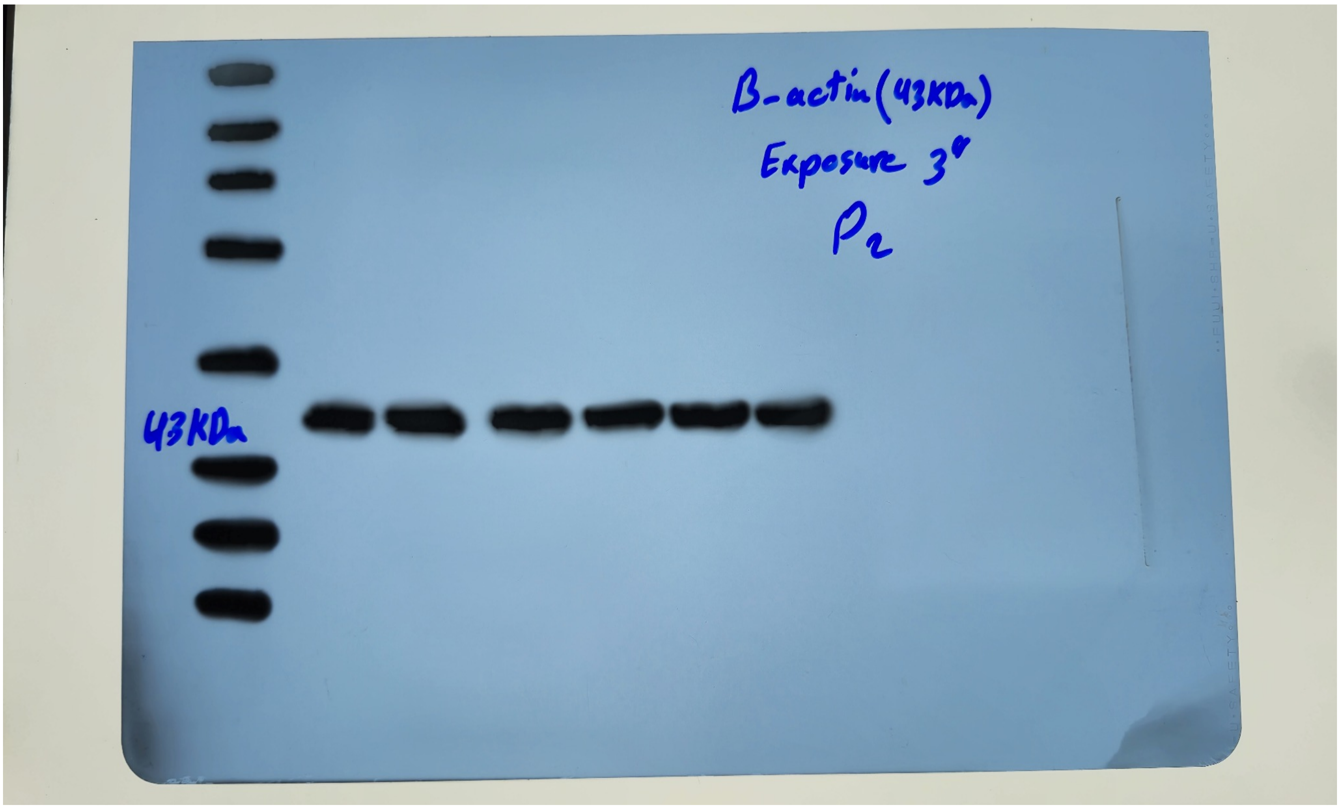

Figure S6. β-actin (43 kDa) – P3 exposure (3 s)

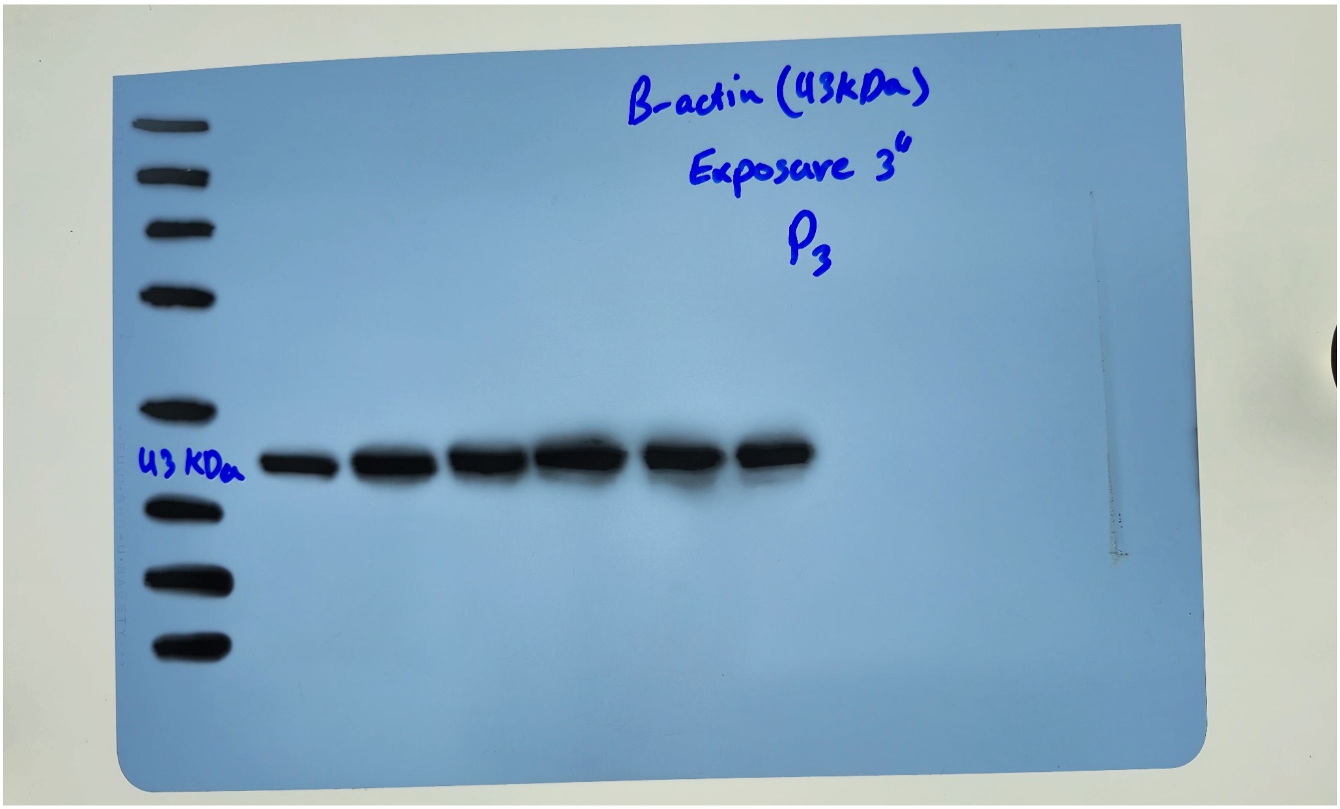

Supplement: Supplementary file 1 — Supplementary Material 1 [file 41598_2026_48488_MOESM1_ESM.pdf]
